# Narrative Review of Immunotherapy in Gastroentero-Pancreatic Neuroendocrine Neoplasms

**DOI:** 10.3390/curroncol30090627

**Published:** 2023-09-21

**Authors:** Jasmeet Kaur, Namrata Vijayvergia

**Affiliations:** Department of Hematology/Oncology, Fox Chase Cancer Center, 333 Cottman Avenue, Philadelphia, PA 19111, USA; jasmeet.kaur@tuhs.temple.edu

**Keywords:** neuroendocrine neoplasm, neuroendocrine carcinoma, immunotherapy, gastroenteropancreatic, pancreatic neuroendocrine tumor

## Abstract

Gastroentero-pancreatic Neuroendocrine Neoplasms (GEP-NENs) are a diverse group of rare tumors that arise from neuroendocrine cells in the gastrointestinal tract and pancreas, and they can vary significantly in terms of clinical behavior and prognosis. Immunotherapy, particularly immune checkpoint inhibitors, has shown remarkable success in various malignancies by harnessing the body’s immune system to target and eliminate cancer cells. Immune checkpoint inhibitor clinical studies in GEP-NENs have yielded promising outcomes, particularly in individuals with advanced and refractory disease. Objective responses and disease stabilization have been observed in some cases, even in those previously unresponsive to traditional treatments like chemotherapy or targeted therapies. However, it’s important to note that the efficacy of immunotherapy in GEP-NENs can vary widely depending on tumor characteristics, the immune microenvironment, and patient factors. As such, identifying predictive biomarkers to select the most suitable patients for immunotherapy remains an ongoing challenge. Immunotherapy has considerable potential for treating GEP-NENs, but research is still in its early stages. Several combinations are being explored to enhance the effectiveness of immunotherapy and improve the outcomes of treatment, such as combining immunotherapy with other targeted therapies or chemotherapy.

## 1. Introduction

Gastro-enteropancreatic neuroendocrine neoplasms (GEP-NEN) are relatively rare tumors, with an incidence of approximately 2.5 to 5 cases per 100,000 people per year [1]. They are more common in adults over the age of 50 and slightly more common in women than men. The incidence of GEP-NEN has been increasing over the past few decades, in part due to improved diagnostic techniques and increased awareness of the disease [1]. GEP-NEN are heterogeneous tumors with a wide range of histopathological features. They can be classified into three main subtypes based on their location: foregut (stomach, duodenum, and pancreas), midgut (small intestine, appendix), and hindgut (colon and rectum). The management of GEP-NEN depends on the location, grade, and stage of the tumor and requires multi-disciplinary input (Table 1) [2]. Surgical resection is the primary treatment for localized tumors, while systemic therapy with somatostatin analogs, chemotherapy, and targeted therapies may be used for advanced or metastatic disease. In addition, peptide receptor radionuclide therapy (PRRT) has emerged as a promising treatment option for GEP-NEN, particularly for those with high levels of somatostatin receptors [2,3]. GEP-NENs represent an intriguing avenue of research for several compelling reasons. The heterogeneous nature and variable clinical behavior of GEP-NENs present unique treatment challenges [4]. It is often difficult to achieve durable responses with conventional therapeutic approaches. Immunotherapy, which targets cancer cells by using the body’s immune system, may be novel and potentially transformative. Several molecular profiles and interactions with the tumor microenvironment suggest that immune interventions might be effective in certain subsets of these tumors [4]. Immunotherapy has largely been successful in treating other forms of cancer, which suggests it can have a similar effect on GEP-NENs [5]. A better understanding of the immunogenicity of GEP-NENs and their response to immune-modulating treatments may hold the key to developing more effective and personalized treatments for this challenging malignancy. Immunotherapy is being actively investigated as a treatment for GEP-NEN [6]. Immune checkpoint inhibitors and adoptive T-cell therapy have shown promising results in preclinical studies and early-phase clinical trials [7]. In this review, we will discuss the role of immunotherapy-based approaches, limitations, and future perspectives in the GEP-NEN.

## 2. Description of the Literature Search

The purpose of this study is to provide a comprehensive and in-depth review of existing literature regarding the tumor microenvironment and immunotherapy with regard to GEP-NENs. To accomplish this, a meticulous and systematic search was conducted on the PubMed database, specifically focusing on articles available in the English language. We aimed to include discussions on immunotherapy in relation to clinical trials involving GEP-NENs in our search. This study aims to provide valuable insights into the intricate interplay between the tumor microenvironment and immunotherapy’s potential as a therapeutic avenue in the field of GEP-NENs by synthesizing and analyzing the wealth of information available in these scholarly resources. Many of the studies analyzed in this review did not distinguish between neuroendocrine carcinoma (NEC) and neuroendocrine tumors (NET). Instead, they collectively considered them neuroendocrine neoplasms (NENs). In our review, we will adopt a similar approach by initially describing NENs as a whole and subsequently providing detailed insights into the specific cohorts selected within the included studies.

## 3. Tumor Microenvironment and Scientific Rationale of Immunotherapy of GEP-NEN

The tumor microenvironment (TME) plays a critical role in the growth and progression of GEP-NEN [8]. The components of the TME in GEP-NEN include stromal cells, immune cells, angiogenic factors, and the Extracellular matrix (ECM), which interact with each other to promote or inhibit tumor growth and metastasis [9]. Immune cells, including T cells, B cells, natural killer (NK) cells, and myeloid-derived suppressor cells (MDSCs) [10,11], can either promote or inhibit tumor growth and metastasis depending on their phenotype and functional status. For example, cytotoxic T cells and NK cells can kill cancer cells, while MDSCs can suppress immune responses and promote tumor growth. The TME in GEP-NEN is highly vascularized, with the production of several angiogenic factors, including vascular endothelial growth factor (VEGF) and platelet-derived growth factor (PDGF), which stimulate endothelial cell proliferation and vessel formation [9,12].

Several studies showed that GEP-NEN expresses immune checkpoint molecules, such as programmed death-ligand 1 (PD-L1), which can suppress the immune response and promote tumor growth [13,14]. In a retrospective study by Bosch et al., tumor specimens of 244 patients with GEP-NEN were identified with PD-1 (program death)/PDL-1 expression and TILs (tumor-infiltrating lymphocytes) [15]. The 244 samples comprised 8.2% grade 3 samples, 34% grade 2 samples, and 57.8% grade 1 samples. The TIL was found to be high (>3 positive lymphocytes) in 47 cases (19.6%). Grade 3 tumors had substantially higher TIL levels than grade 1 and 2 cancers (50% vs. 17.1%; *p* 0.0001). Similarly, 53.8% of grade 3 tumors tested positive for PD-1, compared with 13.7% of grade 1/2 tumors (*p* 0.001) [15]. There was, however, no significant relationship between PDL-1 expression and tumor grading. Low TILs (<3 positive lymphocytes) were associated with a substantially longer overall survival (OS) of 53.9 months (CI 95%: 51.7; 56.1 months) compared with higher TILs, who had a mean OS of 39.4 months (CI 95%: 32.2; 46.6 months; *p* 0.001). High TILs with grade 3 tumors had a worse OS of 8.8 months (CI 95%: 3.9; 13.6 months). PDL-1 and PD-1 expression levels were found to be substantially associated with poor overall survival.

In another study, 102 surgically resected small bowel NETs (neuroendocrine tumors) were examined for tumor-associated immune infiltrates [16]. PDL1 expression was found in more than 1% of tumor cells in 39% of cases and in more than 50% of tumor cells in 14%. In 66% of cases, there was intratumoral infiltration. However, there was no prognostic significance associated with PDL-1 expression or the degree of immune infiltration. Poorly differentiated NECs (neuroendocrine carcinoma) tend to have higher rates of mutations and thus a higher burden of neoantigens, which can make them a more attractive setting to study the role of checkpoint inhibitors (CPIs) [17]. The scientific rationale for the use of immunotherapy in GEP-NEN is based on the expression of immune checkpoint molecules, the presence of TILs, the expression of neoantigens, and the potential for synergy with other treatments [11].

## 4. Clinical Trials of Immune Checkpoint Inhibitors in NENs (Table 1)

Several phase II studies have recently explored single-agent and combination therapy with immune checkpoint inhibitors.

*Monotherapy-Targeting PDL-1 and PD-1:*

***Pembrolizumab***: The Keynote-028 trial tested pembrolizumab in PDL-1-positive solid tumors. The NEN cohort included 25 patients with carcinoid tumors and 16 with pancreatic neuroendocrine tumors (pNET). Patients received pembrolizumab for up to 24 months. Results showed a 6.3% response rate in pNET patients, and 69% experienced treatment-related toxicity, with fatigue and diarrhea being common. Hypothyroidism was the most frequent immune-related adverse event [18].

Keynote-158 was a phase 2 basket study that included patients with disease progression on one or more prior lines of therapy. It enrolled 107 patients with NETs, and out of them, 83 were GEP-NEN [19]. The patients received pembrolizumab 200 mg every 3 weeks for up to 2 years. The combined ORR (Objective Response Rate) in this study was 3.7% (95%CI; 1–9.3), with zero complete responses and 4 partial responses. The median follow-up was 24.2 months, and the duration of response (DOR) was not reached. The median overall survival (OS) was 24.2 months (95% CI; 15.8–32.5). Grade 3 or above toxicities were observed in 21.5% of patients.

Looking specifically at G3 NENs, the joint analysis of two phase-2 open-label trials enrolled 29 patients with previously treated G3 NEN to receive Pembrolizumab 200 mg every 3 weeks [20]. The Ki67 index was less than 50% in 14 patients, and 12 patients had more than 50% of the Ki67 proliferation index. The study demonstrated only one patient with a large cell esophageal neuroendocrine carcinoma had an objective partial response (3.4%, 95%CI; 0.1–17.8%), while six patients had stable disease (20.7%, 95% CI; 7.9–39.7%). The median PFS was 8.9 weeks, and the median OS was 20.4 weeks. No difference in OS or PFS was observed among PDL-1-positive versus negative tumors.

***Spartalizumab:*** The role of Spartalizumab, a PD-L1 inhibitor, in NEN was evaluated in a phase II, multi-center, single-arm [21]. The study enrolled 95 patients with well-differentiated grade 1 or 2 NET, of which 65 patients were of GEP origin and 30 patients had thoracic NET. The study also enrolled 21 patients with poorly differentiated grade 3 GEP-NEN. All the patients had disease progression after prior treatment. The patients in the study received spartalizumab 400 mg every four weeks until disease progression or unacceptable toxicity. Unfortunately, the study did not achieve its primary outcome of ORR > 10%. In the NET group, the overall ORR was 7.4% (95% CI: 3.0, 14.6), with a maximum ORR of 13.7% observed in the thoracic NET. However, the ORR was low in GI NET and pancreatic NET, compared with the overall ORR at 3.1% and 3%, respectively. The ORR in GEP-NEN was 4.8%.

***Toripalimab***: A phase Ib trial evaluated the role of Toripalimab in patients with NEN [22]. The study enrolled 40 patients, with eight with WD-NET, of which seven patients had GEP origin, and 32 patients with PD-NEN, of which 25 had GEP origin. The PDL-1 expression of >1% was seen in 14 patients, of whom three had WD-NET and 11 had PD-NEN. The study’s primary endpoint was ORR, and the secondary endpoint was DCR, PFS, and OS [22]. The overall ORR of the 40 patients enrolled in the study was 20%. However, patients with PDL-1 expression >10% and high tumor mutation burden (TMB) had significantly higher ORRs compared with patients with PDL-1 expression below 1% (50.0% vs. 10.7%, *p* = 0.019) or low TMB (75.0% vs. 16.1%, *p* = 0.03).

***Avelumab:*** Avelumab, a PDL-1 inhibitor, was first studied in a phase II multicenter study that included 29 patients, of whom 16 had NEC grade 3 and 11 had NET grade tumors who progressed after first-line chemotherapy [23]. The study achieved the primary outcome of a disease control rate (DCR) of 32% after eight weeks, but the median OS was only 4.2 months.

The results of two-phase clinical trials, NET001 and NET002, were combined to evaluate the response of Avelumab in high-grade NEN who had previously received systemic therapy [24]. The study included 27 patients, of whom 21 were GEP, with a median Ki67 index of 35%. The study did not achieve the primary endpoint of ORR. The DCR at six months was 21%, and 33% of patients had stable disease.

## 5. Combination Therapy (Table 1)

- *Dual Immune checkpoint inhibitors (ICI)* (-Targeting CTLA-4 + PDL-1/PD-1)

Dual ICI has shown promising results in metastatic hepatocellular carcinoma, melanoma, lung cancer, mesothelioma, and renal cell cancer [25,26,27]. Based on these results, several clinical trials in NEN have been conducted using dual ICI. The results are somewhat conflicting and highlight the importance of a well-designed prospective study to define its role. (Table 1)

***Nivolumab plus Ipilimumab***: The DART trial, a phase II clinical trial that included multiple tumor cohorts, studied the combination of anti-CTLA-4 (cytotoxic T-lymphocytes associated-4) and anti-PD-1 [28]. In the NEN cohort, 32 patients received ipilimumab in combination with nivolumab, of which 25 were gastrointestinal NEN (19 small intestines, 6 stomachs). The overall ORR was 25% (95% CI; 13–42%). However, patients with high-grade NEN had a significantly higher ORR of 44% (95% CI; 22–69%), while in low-grade NEN, it was 0%. In addition, the overall PFS at six months was 31% higher in high-grade NEN at 44% and 14% in low-grade NEN. The median OS is 11 months (95% CI 6-NE). The lack of central confirmation of grading makes it difficult to define the role or setting of this combination in NENs.

A similar study, CA209-538, evaluated ipilimumab in conjunction with nivolumab in 29 patients with advanced NEN [29]. Three patients had low-grade NEN, 13 patients had intermediate-grade NEN, and the remaining 13 had high-grade NEN. The overall ORR was 24%, with a clinical benefit rate of 72%. The pancreatic NEN cohort had an ORR of 43% (3 of 7 patients in the pancreatic NEN cohort), 2 patients had grade 3 NEN, and one patient had small cell carcinoma. The median PFS was 4.8 months (95% CI; 2.7–10.5), and the median OS was 14.8 months (95% CI; 4.1–21.3). Grade 3 or more AE was observed in 34% of patients, and immune-related AE was seen in 66%.

***Durvalumab plus Tremelimumab***: The DUNE trial was a phase 2 multi-cohort trial that enrolled 123 patients with GEP and lung NEN patients [30]. There are four cohorts in the study. Cohort 1 enrolled 27 patients with typical and atypical lung carcinoid; cohort 2 enrolled 31 patients with grade 1/2 gastrointestinal NEN; cohort 3 enrolled 41 patients with grade 3/4 pancreatic NEN; and cohort 4 enrolled 33 patients with grade 3 GEP-NEN [30,31]. The patients received a combination of Durvalumab and Tremelimumab. The overall clinical benefit rate was 56.1% (95% CI: 47.3–64.6). Accordingly, cohort 1 showed a 9-month CBR of 25.9% (95% CI: 12.4–44.3), cohort 2 showed 35.5% (95% CI: 20.5–51.0), cohort 3 showed 25% (95% CI: 12.6–41.7), and cohort 4 showed 6.1% (95% CI: 1.3–18.1). The study achieved the primary endpoint of OS at nine months for grade 3 GEP-NEN, which was 36.1%. However, the ORR was disappointing at 9.1%. The mOS was not reached for cohort 1, but for cohorts 2 to 4, it was 29.5 m (19.6–39.4), 23.8 m (16.4–31.2), and 5.9 m (CI: 2–9.7), respectively. There was no difference in outcomes seen based on PDL-1 expression in these patients. The most common AE was liver toxicity, diarrhea, fatigue, and vomiting.

- *Combination of ICI with TKI*

***Atezolizumab plus Bevacizumab***: The combination of Bevacizumab and Atezolizumab was studied in 20 patients with PNET and 20 patients with extrapancreatic (ep) NET. The study demonstrated ORR in PNET 20% (95% CI, 5.7–43.7%) and 15% in ep NET (95% CI, 3.2–37.9%) [32].

***Pembrolizumab plus Lenvatinib***: A phase II study evaluated the combination of Pembrolizumab and Lenvatinib in 20 patients with GI and thoracic NET [33]. Unfortunately, the study did not meet the primary outcome of achieving ORR in four patients. However, 12 patients (60%) had grade 3 adverse effects, and 14 patients required either dose reduction or medication discontinuation.

- *Combination of ICI with somatostatin analogs*

***Pembrolizumb plus Lanreotide***: A phase II trial evaluated the role of a combination of pembrolizumab and lanreotide in GEP-NEN, who progressed on somatostatin analogs [34]. The study included 22 patients with a median Ki-67 index of 5%. Although the primary endpoint was ORR, the best response observed was stable disease in 39%, with 52% of patients having progressive disease. The median PFS was 5.4 months (95% CI; 1.7–8.3), and the median OS was 15 months (NR).

- *Combination of ICI with chemotherapy in G3 NEN*

A phase II open-label, non-randomized clinical trial comparing the role of combining chemotherapy with pembrolizumab versus pembrolizumab alone in high-grade malignant NEN enrolled 36 patients, and there were 14 patients in the pembrolizumab group versus 22 patients in the combination group [35]. The primary objective of ORR was seen in 7% (0.02 to 33.9%) of the pembrolizumab alone group versus 5% in the combination group (0 to 22.8%).

Temozolomide (TMZ) has been demonstrated to have immunomodulatory effects on lymphoid cells in patients with melanoma [36]. A phase 2 clinical trial enrolled 28 patients with NEN, out of whom 20 had NET and 8 had NEC [37]. There were 13 patients who had GEP-NEN. The patients received combination treatment with TMZ and nivolumab. The ORR was 32.1% (95% CI: 15.9–52.4) [37]. Blood immune cell profiling after 2 weeks of treatment showed an increase in circulating CD8+ T cells (27.9% 13.4% vs. 31.7% 14.6%; *p* = 0.03) and a decrease in CD4+ T cells (59.6% 13.1% vs. 56.5% 13.0%; *p* = 0.001) comparing it with the screening sample.

## 6. Current Treatment Paradigm with NCCN Recommendations (Figure 1)

The National Comprehensive Cancer Network (NCCN) provides guidelines for the diagnosis, treatment, and follow-up of GEP-NEN. The NCCN guidelines are based on the latest available evidence from clinical trials, expert opinion, and consensus [2]. The NCCN guidelines for the treatment of GEP-NEN are stratified based on tumor grade, site of origin, and the presence or absence of metastases.

**Figure 1:**
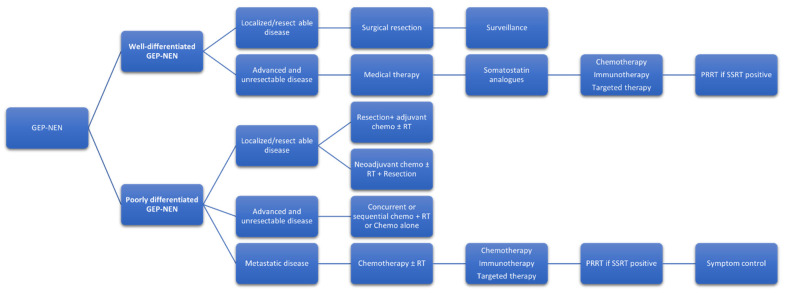
**Current treatment paradigm Gastro-enteropancreatic Neuroendocrine neoplasm.** GEP-NEN: Gastro-enteropancreatic neuroendocrine neoplasm; RT: Radiotherapy; SSRT: PRRT: peptide receptor radionuclide therapy; SSRT: somatostatin receptor imaging.

For patients with well-differentiated, localized GEP-NEN, surgical resection is the recommended treatment approach. Observation may be considered in selected patients with small, asymptomatic tumors. For patients with well-differentiated, metastatic GEP-NEN, somatostatin analogs (SSAs) are recommended as first-line therapy, regardless of the site of origin.

SSAs are effective in controlling symptoms associated with hormonal hypersecretion and may also have antitumor effects. Other treatment options include peptide receptor radionuclide therapy (PRRT), targeted therapy with everolimus or sunitinib, and chemotherapy. For patients with poorly differentiated, metastatic GEP-NEN, chemotherapy is the recommended first-line therapy. Platinum-based regimens, such as cisplatin and etoposide, are the preferred chemotherapy options [2]. PRRT may be considered in selected patients with high somatostatin receptor expression. The updated NCCN 2022 version 2 guidelines recommend a combination of ipilimumab and nivolumab as a category 2B recommendation for patients with the metastatic progressive disease for NEC and well-differentiated grade 3 NET [38].

## 7. Future Direction

The future direction for GEP-NEN research and treatment involves precision medicine, immunotherapy, combination therapy, patient-reported outcomes, and novel imaging techniques. As the field continues to evolve, these advances may improve outcomes and quality of life for patients with GEP-NEN. Advances in molecular profiling techniques have allowed for a better understanding of the genetic and molecular characteristics of GEP-NEN. Concentrating our efforts to identify biomarkers or responses to immunotherapeutic agents will be of prime importance. Similarly, rational combinations of immunotherapeutic agents with existing and novel therapies are warranted to improve outcomes and harness the power of immunotherapy in this hard-to-treat disease. As displayed in Table 1, multiple ongoing and planned studies are evaluating this.

Chemotherapy can induce immunogenic cell death, which can promote the release of antigens and activate the immune response. Combining immunotherapy with chemotherapy may enhance the antitumor immune response and improve treatment outcomes. There has been significant advancement in the treatment of neuroendocrine carcinomas (NECs), with emerging evidence supporting a combination of chemotherapy and immunotherapy as an effective strategy. There are many ongoing trials evaluating the role of the combination of chemotherapy with immunotherapy, e.g., the phase II trial NCT03980925 evaluating the role of the combination of nivolumab with carboplatin and etoposide and the NCT01174121 trial studying the combination of chemotherapy with Pembrolizumab, Interleukin-2 with Tumor infiltrating (TIL) cells. S2102 is an ongoing national phase II/III trial that compares atezolizumab in combination with standard chemotherapy (cisplatin or carboplatin + etoposide) versus standard therapy alone for the treatment of poorly differentiated extrapulmonary NECs, with an accrual target of 189 patients. This study will likely provide practice-defining guidance on the role of adding checkpoint inhibitor therapy in a frontline setting for NECs (NCT05058651). Another ongoing trial is evaluating the role of Atezolizumab as a standard chemotherapy treatment for advanced or metastatic extrapulmonary neuroendocrine carcinomas (NCT05058651) (Table 2).

The combination of ICI with TKI has shown promising results in other solid malignancies, including renal cell carcinoma, hepatocellular carcinoma, melanoma, and bladder cancer [39,40,41]. In addition, there are ongoing trials in neuroendocrine tumors to evaluate the role of combining ICI with TKI. Apart from those mentioned in earlier sections, more studies are ongoing, including a combination of Tislelizumab plus Surufatinib (NCT04579757) and Avelumab plus Regorafenib (NCT03475953). (Table 2).

- *Combination of ICI with lutathera*

177Lu-DOTATATE is approved for somatostatin-positive advanced GEP-NEN tumors [42]. The synergistic effect of radiation and immunotherapy is being studied in this phase 2 trial, NCT04525638, combining 177Lu-DOTATATE with Nivolumab.

- *Vaccine and CART cell therapy*

Several studies have shown that GEP-NEN has high levels of TILs, particularly in the metastatic setting [11,43]. This suggests that GEP-NEN may be particularly susceptible to immunotherapy approaches like vaccines and adoptive cell therapy. There are ongoing trials evaluating the role of vaccine therapy in NEN tumors, including = SVN53-67/M57-KLH Peptide Vaccine (NCT03879694; phase I: NCT02455557; phase II). Adoptive cell therapy is another approach currently being studied in advanced GEP-NEN. In a preclinical study, the CDH17-expressing GEP-NEN tumor showed improved outcomes with CDH17 CART cell therapy [44]. Similarly, somatostatin receptor type 2 (SSRT2) CART cell therapy is being developed and has shown significant anti-tumor activity in preclinical studies [45].

**Table 1:** Clinical Trials in Gastro-enteropancreatic Neuroendocrine neoplasm.

Trial	Disease Site	Grade	Phase, Study Design	No.	Drug	ORR (95% CI)	PFS (95% CI)	OS (95% CI)	Toxicities Grade > 3
**Monotherapy -Targeting PDL-1 and PD-1**									
Mehnert et al 2020 [18] Keynote-028 NCT02054806	Carcinoid tumor	NA	I, open label, single group assignment	25	Pembrolizumab 10 mg/Kg every 2 weeks	12 (2.5–31.2)	5.6 m (3.5–10.7)	NA	4%
	pNET	NA		16		6.3% (0.2–30.2)	4.5 m (3.6–8.3)	NA	6.3%
Strosberg, et al, 2020 [19] Keynote -158 NCT02628067	NET	1,2	II, open label, non-RCT	24	Pembrolizumab 200 mg every 3 weeks	3.7% (1–9.3)	4.1 m (3.5–5.4)	24.2 (15.8–32.5)	21.5%
	GEP-NEN			83					
Yao, et al 2021 [21] NCT02955069	NET	1,2	II, open label, single group assignment	95	Spartalizumab 400 mg every 4 weeks	7.4% (3.0–14.6),	19.5% (12 m PFS)	73.5% at 12 months (63–81.4)	20 (21.1%)
	GEP-NEC	3		21		4.8% (0.1–23.8)	0% (12 m PFS)	19.1% at 12 months (4.8–40.6)	4 (19%);
Lu et al 2020 [22] NCT03167853	WD-NEN	2,3	Ib, open label, single group assignment	8	Toripalimab 3 mg/kg every 2 weeks	25%	2.5 (1.9–3.1)	7.8 (5–10.8)	11 (27.5%)
	PD-NEN	3		32		18.7%			
Fottner et al 2019 [23] AVENEC NCT03352934	GEP	3	II, open label, single group assignment	27	Avelumab 10 mg/kg every 2 weeks	--	3.3 m (1.2–24.6)	14.2 m	10%
**Dual Immune check point inhibitors (-Targeting CTLA-4 + PDL-1/PD-1)**									
Patel et al 2020 [28] DART trial NCT02834013	NEN	1,2,3	II	32	Nivolumab 240 mg every 2 weeks plus Ipilimumab 1 mg every 6 weeks	25% (13–42)	6% at 6 months (19–52%)	11 m (6-NE)	16 (50%)
Klein et al 2020 [29] CA209-538 NCT02923934	NEN	1,2,3	II	29	Nivolumab 3 mg/kg plus Ipilimumab 1 mg/kg every 3 weeks for four doses followed by Nivolumab 3 mg/kg every 2 weeks upto 96 weeks	24%	4.8 m (2.7–10.5)	14.8 M (4.1–21.3)	10 (34%)
Girard et al., 2021 [46] NCT03591731	GEPNET and Lung NEC (PD)	NEC	II	170	Nivolumab plus Ipilimumab	14.9% (8.2–24.2)	1.9 m (1.6–2.1)	7.2 m (3.7–14.1)	--
		NEC			Nivolumab	7.2% (2.7–15.1)	1.8 m (1.7–2.0)	5.8 m (3.3–7.6)	--
Capdevila et al 2020 [30,31] DUNE trial NCT03095274	Lung NEN	1,2	II	27	Durvalumab 20 mg/kg every 4 weeks plus Tremelimumab 1 mg/Kg every 4 weeks	11.1%	5.6 m (4.9–6.2)	NR (0.3–41.3)	12.2%
	GI-NET	1,2		31		0%	5.8 (3.1–8.5)	29.5 (19.6–39.4)	
	p-NET	1,2		32		6.3%	5.5 (2.4–8.7)	23.8 (16.4–31.2)	
	GEP-NEN	3		33		9.1%	2.4 (2.1–2.8)	9 months OS 36.1% (19.6–52.6)	
**ICI combined with TKI**									
Halperin et al. 2022 [32] NCT03074513	p-NET	1,2	II, open label, single group assignment	20	Atezolizumab 1200 mg plus Bevacizumab 15 mg/kg every 3 weeks	20% (5.7–43.7)	14.9 (4.4–32.0)	30.1 m (17.7 m-NR)	--
	Ep-NET			20		15% (3.2–37.9)	14.2 (10.2–19.6)	NR	--
Al-Toubah et al. 2022 [33] NCT03290079	NET	--	II, open label, single group assignment	20	Pembrolizumab 200 mg every 3 weeks plus Lenvatinib 20 mg daily	10%	10 m (5.9–14.1 m)	--	12 (60%)
Morse et al 2021 [34] PLANET trial NCT03043664	GEP-NEN	--	II, open label, single group assignment	22	Pembrolizumb 200 mg every 3 weeks plus Lanreotide 90 mg every 3 weeks	39%	5.4 m (1.7–8.3)	15 m (NR)	--
Raj et al. 2023 [35] NCT03136055	NEN	--	II, open label, Non-RCT	14	Pembrolizumab 200 mg every 3 weeks for 24 months or 35 administrations	7% (0.02–33.9)	1.8 m (1.7–21.4),	7.8 (3.1-NR)	2 (14%)
		--		22	Pembrolizumab 200 mg every 3 weeks for 24 months or 35 administrations plus Irinotecan 125 mg/m^2^ day 1 and day 8 every 3 weeks plus paclitaxel day 1, 8 and 15 every 3 weeks	5% (0–22.8)	2.0 m (1.9–3.4),	4.8 (4.1–8.2)	10 (45%)

GEP-NEN: Gastro-enteropancreatic Neuroendocrine neoplasm; NEN: neuroendocrine neoplasm; NET: neuroendocrine tumor; NEC: neuroendocrine carcinoma; p-NET: pancreatic neuroendocrine tumor; ep-NET: extra pancreatic neuroendocrine tumor; WD-NEN; well differentiated neuroendocrine neoplasm; PD-NEN: poorly differentiated neuroendocrine neoplasm; GI-NET: gastrointestinal neuroendocrine tumor; ORR: overall response rate; PFS: progression free survival; OS: overall survival; PDL-1: program death receptor ligand; PD-1 program death receptor- 1; ICI: immune check point inhibitor; TKI: tyrosine kinase; CTLA-4: cytotoxic T- lymphocytes associated-4. RCT: randomized control trial.

**Table 2:** Ongoing trial in Gastro-enteropancreatic Neuroendocrine neoplasm.

Trial	Disease Site	Phase	No.	Drug	Primary Outcome	Secondary Outcome	Result/Status
NCT01174121 Parallel arm design	Metastatic cancer (NET)	2	332	TIL, Pembro, Aldesleuki + Chemo	Response rate	Safety and efficacy	Accruing
NCT03980925; Single group	GEPNET	2	38	Nivo + platinum doublet	OS at 12 months	PFS, ORR	Accruing
NCT03290079; Single group	NET	2	28	Pembro + Lenvatinib	ORR	DOR, PFS, OS	Active not accruing
NCT03475953; Sequential assignment	Solid tumor (GEPNET)	2	482	Regorafenib + Avelumab	RP2D, antitumor activity of regorafenib	MTD, DLT	Accruing
NCT04579757	Solid tumor (NET)	2	135	Surufatinib and Tislelizumab	ORR, dose limiting toxicity	PFS	Active not accruing
NCT05058651	Extrapulmonary NEC	2/3	189	Atezolizumab, etoposide, platinum	OS	PFS, ORR, DOR	Accruing
NCT04079712	NET	2	30	Cabozantinib +Nivolumab +Ipilimumab	ORR	PFS, AE	Active not accruing
NCT04525638	NET (G3 WD)	2	30	177Lu-DOTATATE and Nivolumab	ORR	PFS, OS, AE	Accruing
NCT02749331 RADNET	NET	1/2a	35	Recombinant Adenovirus AdVince	AE	PFS, changes in tumor size,	Accruing
NCT03879694	NET (metastatic)	1	14	Survivin Long Peptide Vaccine (SurVaxM)	AE	TTP, DOR, ORR	Accruing

GEP-NEN: Gastro-enteropancreatic Neuroendocrine neoplasm; NEN: neuroendocrine neoplasm; NET: neuroendocrine tumor; NEC: neuroendocrine carcinoma; WD-NEN; well differentiated neuroendocrine neoplasm; PD-NEN: poorly differentiated neuroendocrine neoplasm; GI-NET: gastrointestinal neuroendocrine tumor; ORR: overall response rate; PFS: progression free survival; OS: overall survival; DOR: duration of response; AE: adverse effect; MTD: maximum tolerated dose; DLT: dose limiting toxicity; TTP: time to progression.

## 8. Scientific Limitations

While immunotherapy holds immense promise as a transformative treatment approach, it is essential to acknowledge its limitations, particularly in the context of GEP-NENs. In GEP-NENs, heterogeneity affects immune responsiveness, which can vary between tumor subtypes and patients, preventing consistent therapeutic outcomes. The immunosuppressive microenvironment commonly observed in GEP-NENs might also counteract the intended effects of immunotherapy, reducing its efficacy. Additionally, the relatively low mutational burden in GEP-NENs compared with other malignancies may reduce the availability of neoantigens, which are crucial for immune recognition. The identification of biomarkers that reliably predict which patients will respond favorably to immunotherapy is another challenge. A limited number of clinical trials have been conducted specifically on GEP-NENs, which may make it difficult to develop tailored immunotherapy strategies for this cancer subtype. Despite these challenges, ongoing research efforts are actively working to address these limitations and unlock the full potential of immunotherapy for GEP-NENs, ultimately paving the way for improved patient outcomes.

## 9. Conclusions

ICIs have shown promising results in the treatment of many malignancies. However, the role of immunotherapy in GEP-NENs is currently being investigated, and its efficacy may differ depending on tumor grade, stage, and immune microenvironment.

With the exception of the DART trial, which showed some promise, the overall success of immunotherapy in GEP-NENs has been limited. The tumor grade is one factor that may influence the immunotherapy response in GEP-NENs. G3 neuroendocrine carcinomas (NECs), which are high-grade and aggressive tumors, have been shown to respond better to immunotherapy than lower-grade GEP-NECs. This could be because G3 NECs have a larger mutational burden and higher expression of immune checkpoint proteins, making them more vulnerable to immune regulation. However, despite these findings, immunotherapy response rates in GEP-NENs, particularly G3 NECs, have been generally modest. This emphasizes the need for novel treatments as well as continuous research efforts in this area. New approaches are being explored to improve GEP-NEN patient outcomes. In addition, different immunotherapeutic agents, combinations of agents, and predictive biomarkers can be identified to determine which patients will benefit most from immunotherapy. Moreover, strategies are being developed by understanding the underlying mechanisms to overcome resistance to immunotherapy in GEP-NENs.

Overall, while immunotherapy has had limited success in GEP-NENs thus far, current research and the study of innovative therapeutic options show promise for improving outcomes in this challenging disease. Efforts are being made to discover appropriate patient selection criteria, create effective combination techniques, and deepen our understanding of the immunobiology of GEP-NENs in order to improve treatment strategies for patients in need.

## Data Availability

Not applicable.
